# Beyond the “weakness of the state”: Canada’s intervention in post-agreement Colombia

**DOI:** 10.1177/00207020221135370

**Published:** 2022-10-18

**Authors:** Marc-André Anzueto, Etienne Roy Grégoire, Philippe Dufort

**Affiliations:** 59310 Université du Québec en Outaouais, Gatineau, QC, Canada; Université du Québec à Chicoutimi, Chicoutimi, QC, Canada; 10064 Saint Paul University, Ottawa, ON, Canada

**Keywords:** business and human rights, Colombia, Canada, rule of law, cooperation, counterinsurgency, transitional justice, extractivism, democracy

## Abstract

During the 2021 mass protests in Colombia, and while international calls for the Colombian government to respect human rights were intensifying, Canada’s position remained somewhat ambiguous. Part of Canada’s ambiguity can be explained by a simplistic characterization of Colombia as a “weak state.” This article assesses Canada’s bilateral relationship by historizing the development of Colombia’s governance in the key overlapping sectors of security, human rights, and natural resources. From extensive fieldwork, we distinguish two competing rationalities based on the articulation of the notions of “conflict” and “dissent” with the notion of the “rule of law.” We believe that Canada’s bilateral relation with Colombia in the last decades has overlooked the contradictions that exist between democratizing rationalities and antipolitical rationalities. As a result, Canada’s foreign policy has been based on an overly simplistic conception of the relationship between development, security, and the rule of law.

On 28 April 2021, a national strike began in Colombia. In the context of an ongoing economic crisis, broad social dissatisfaction, and the COVID-19 pandemic, Colombian citizens were initially protesting President Iván Duque’s tax reform plan. Serious human rights abuses were committed by Colombian security forces against protesters, with reports of forced disappearances, gender-based violence, and ethnic-racial violence in several Colombian cities.^1^ As international calls for the Colombian government to respect human rights were growing more persistent, Canada’s position remained somewhat ambiguous. In his only statement on the situation on May 9, the Canadian minister of foreign affairs, the Honourable Marc Garneau, expressed concern for “the disproportionate use of force by security forces,” but tempered his remarks by condemning “acts of vandalism and attacks directed against public officials.”^2^ This tepid stance attracted criticism and raised questions regarding Canada’s foreign policy objectives in Colombia.^3^

At least part of the ambiguity in Canada’s response can be explained by its overly simplistic characterization of Colombia as a State to be “strengthened.” Indeed, while the academic fascination with defining “weak” or “failed” States has waned, these concepts are still used with regards to Colombia,^4^ and remain widely operational in international policy discourse.^5^ Notwithstanding the varied and sometimes contradictory definitions, policies are routinely justified by “fragile state” narratives aimed at “strengthening” different aspects of a nation’s governance and institutions.

Canada’s bilateral relationship with Colombia^6^ is no exception. Indeed, from 2018 to 2020, Canadian official reports on human rights and free trade with Colombia refer to “weak rule of law” as a significant challenge for the peace process and improving human rights protection.^7^ Regardless of this longstanding approach, however, assigning “weakness” to Colombia’s political structures and institutions is too often a blunt process that overlooks how different historical processes “weaken” some institutions while “strengthening” others. Thus, policies aimed at “strenghtening the Colombian State” without specifying institutions or modalities can be called into question.

This article will assess the Canada-Colombia bilateral relationship using a conceptual framework that historizes the development of Colombia’s governance in the key overlapping sectors of security, human rights, and natural resources. To do so, we problematize the coexistence and/or struggles between different rationalities, or “regimes of reason,” in Colombia. These different regimes of reason—which are not necessarily consistent with the different options and ideologies represented in Colombia’s party system—are expressed in various institutions through overlapping and competing discourses, doctrines, mobilizations, cosmovisions, policies, jurisprudences, techniques, and norms.

We begin by presenting our theoretical framework, which draws on the Foucauldian notion of governmentality and the heterodox political economy notion of structural power. We then describe how, building on the ambiguity of the notion of “rule of law,” a continuum of peace, human rights, and development has been constructed within the Canada-Colombia bilateral relationship. We illustrate our argument with an examination of Canadian government support for the work of Lawyers Without Borders Canada (LWBC) in Colombia.

Based on a review of primary and secondary literature and insights obtained through 15 years of ongoing practice and fieldwork, we contrast this hegemonic construction with the specific rationalities underlying two interrelated fields structurally linked to Colombia’s human rights landscape: (a) counterinsurgency practices and transitional justice; and (b) territorial organization and natural resource management. The competing rationalities of these two fields can be respectively described as “democratic” and “antipolitical.” Indeed, these rationalities assume very different articulations between the notion of “conflict” or “dissent,” and the notion of the “rule of law.”

In sum, our argument is the following: Canada’s overall foreign policy toward Colombia has been based on an overly simplistic conception of the relationship between development, security, and the rule of law. Overlooking the contradictions that exist between democratizing rationalities and antipolitical rationalities in Colombia, interventions have often been misguided, favouring regressive dynamics. Following the peace agreement with the Revolutionary Armed Forces of Colombia—People’s Army (FARC-EP) on 24 November 2016, the ambiguity in Canada's approach should be abandoned if Canada is to support a genuine democratic transition in post-agreement Colombia.^8^

## Theoretical framework

As a type of assistance grounded in the post–Cold War environment, “security humanitarianism has become increasingly central to Canada’s relationship with the world.”^9^ In the wake of 9/11, a global security agenda aimed at fixing “failed” states emerged among Western countries and international organizations;^10^ the concept of “weak” or “fragile” states structured the political lexicon on peace, development, and security of Canada’s development assistance programs.^11^ These terms, however, amalgamate diverse states into an oversimplified, homogenous group toward which aid donors “react in a formulaic manner to enhance state capacity—even though these countries drastically differ in terms of security, capacity and legitimacy.”^12^

For instance, the 2005 Fragile States Index (FSI) Annual Report^13^ published by the American magazine *Foreign Affairs* stated that “instability takes the form of episodic fighting, drug mafias, or warlords dominating large swaths of territory (as in Afghanistan, Colombia, and Somalia);”^14^ a definition that, prima facie, equates Colombia’s situation to those of nations with vastly different contexts. Despite numerous criticisms related to defining, identifying, and measuring “weak,” “failed,” and “fragile” states,^15^ these have been recurring adjectives in Canadian international cooperation with Colombia, both before and after the 2016 peace agreements were signed.

Central to the concept’s limitations is the implicit assumption of the “essence” of a state from which one could deductively derive an assessment of a given state’s strength and, a fortiori, propose solutions to its weaknesses.^16^ However, as Michel Foucault reminds us:

The State has no fundamental essence. [It] is nothing other than the effect […] of perpetual processes of “statizations,” of incessant transactions which modify, displace, upset, insidiously modify […] the sources of funding, the modes of investment, the decision-making centres, the forms and types of control, the relationships between local powers, central authority, etc. […] The State is nothing other than the mobile effect of a system of multiple governmentalities (our translation).^17^

In this sense, “governmentality” refers to the different rationalities, or regimes of reason—ways of thinking and ways of feeling—through which different conducts are promoted or regulated and different individuals empowered, disciplined, constrained, or coerced.^18^ These regimes of reason are an integral part of what heterodox political economy calls “structural power.”^19^

Canada’s approach to its bilateral relations with Colombia in the area of security and governance exemplifies this point. David Chandler defines “security governance interventions” as “the projections of external power in or over another state in order to direct or influence the security behaviour of actors within that state.”^20^ In that regard, it “is not only power that is projected but a certain framework of ideas and values and political purpose.”^21^ As we argue below, the projection of coherence between the securitization of Canadian economic interests, justice, and human rights is central to Canada’s policies promoting the rule of law in Colombia. The “whole-of-government approach,” first articulated in the context of the war in Afghanistan, thus constructs a conceptual coherence between diplomacy, defense, development, and commerce.^22^

We agree with scholars who have identified that ambiguous definitions of the rule of law are a condition of possibility for projecting policy coherence.^23^ In fact, coherence with other foreign policy objectives has been an issue since the beginning of Canadian foreign aid during the Cold War.^24^ In keeping with the debate between realism and idealism in the field of International Relations, the debate on Canadian aid has been largely dominated by a conceptual dichotomy between ethical and self-interested motives.^25^ As David Black argues, however, “a more nuanced understanding of the relationship between ethics and interests” is needed to “better captur[e] the dilemmas and ambiguities of ethical purpose.”^26^ Indeed, Canada’s aid to Latin America has been historically shaped by “the tension between relatively strong Canadian commercial interests in the region and pressures from highly mobilized civil society.”^27^

## Canadian intervention and the hegemonic construction of a peace, human rights, and development continuum

According to the Canadian government, the goal in applying a “whole-of-government approach” to its bilateral relations with Colombia is simultaneously to “[expand] trade and investment” while maintaining a “frank dialogue on human rights” and supporting “Colombia’s justice, security and peace-building efforts.”^28^ As highlighted in a comparative study of Canada’s interventions in post-conflict Guatemala and Colombia, “strengthening the rule of law” is frequently mobilized as a way to reconcile policy objectives that are often incompatible when applied on the ground: promoting human rights, promoting regional security, and promoting investments.^29^ The notion also has the advantage of appealing to the various audiences and actors interested in Canada’s foreign policy, including civil society organizations and the business community.^30^

The Colombian extractive sector illustrates both the imperative to reconcile these policy objectives and the difficulties involved. Canadian foreign direct investment (FDI) in the mining sector has represented a significant proportion of Colombia’s gross domestic product (GDP) for the past decade.^31^ According to Natural Resources Canada, Canadian mining assets in Colombia totalled $837 million among twenty-eight companies in 2020.^32^ The impact of Canadian mining activities in Colombia has generated discussion on how to hold corporations and States accountable for human rights and environmental abuses. According to a report by the Justice and Corporate Accountability Project (JCAP), the deaths of six individuals can be linked to the operations of Canadian mining companies in Colombia between 2000 and 2015.^33^

Meanwhile, Canada has consistently used ODA to promote the extractive sector. Between 2012 and 2017, Colombia was Canada’s fourth largest recipient of aid linked to mineral resources and the mining sector, following Indonesia, Tanzania, and Peru.^34^

The rest of this section analyzes the work of Lawyers Without Borders Canada to illustrate both the Colombian context and the hegemonic construction of a *continuum* between Canadian economic interest, justice and human rights.

### Contradictions between human rights and trade objectives

The work of LWBC in Colombia illustrates how best to navigate the Canadian government’s competiting priorities in fragile and conflict-affected states (FCAS).^35^ As previously mentioned, the mining sector in Colombia has enjoyed considerable support from the Canadian government for more than two decades despite its negative impacts on Indigenous, peasant, and afro-descendant populations. Paradoxically, these same communities are the main beneficiaries of Canada’s support for peace, human rights, and transitional justice in Colombia, especially through LWBC.^36^ For instance, between 2003 and 2013, LWBC collaborated with the lawyers’ collective *Colectivo de Abogados José Alvear Restrepo* (CAJAR) on issues related to counterinsurgency practices and transitional justice. During the second phase of the project, “Access to Justice for Indigenous Communities and Other Victims of the Conflict,” from 2010 to 2013, LWBC supported the National Indigenous Organization of Colombia (*Organización Nacional Indígena de Colombia* – ONIC) litigation efforts against land exploitation. Table 1

**Table 1.** Canada’s whole-of-government approach in Colombia (2003–2005).

Governing party	Context in Colombia	Canadian “security governance interventions”
Liberal government of Paul Martin (2003–2006)	Presidency of Álvaro Uribe (2002–2010)	The Stabilization and Reconstruction Task Force (START) and the Global Peace and Security Fund (GPSF) are launched in 2005.
	Paramilitary demobilization process launched by President Uribe and the Justice and Peace Law of Colombia or Law 975 of 2005.	Beginning of LWBC activities in Colombia (2003–2005) and first projects funded by the Canadian International Development Agency (CIDA).
	Attacks against lawyers helping victims of the armed conflict, especially women, Indigenous, and afro-Colombians.	In 2005, Colombia is identified as a “perpetual” priority country by START-GPSF.

In 2003, LWBC launched their first international cooperation missions in “weak and fragile” states such as Afghanistan, Colombia, Nigeria, and Sierra Leone.^37^ Appearing before the Standing Committee on International Trade on 26 May 2008, LWBC’s director general, Pascal Paradis, described Lawyers Without Borders as:

[…] an organization that contributes to the defense and promotion of human rights, the fight against impunity, holding fair and impartial trials and respect for the rule of law in various countries in crisis, developing countries or so-called fragile countries (our translation).^38^

LWBC received its first grant from the Canadian International Development Agency (CIDA) in 2004–2005 for a training and protection program for Colombian defence lawyers. This collaboration with the Canadian government coincided with the creation of the Stabilization and Reconstruction Task Force (START) and the Global Peace and Security Fund (GPSF) in 2005. The Government of Canada created them “with a mandate to advance Canada’s foreign policy priorities of addressing international security challenges and promoting the Canadian values of freedom, democracy, human rights, and rule of law in fragile and conflict-affected states.”^39^

At that time, Canada’s Liberal government “promoted neo-liberal reforms and regional free trade agreements, [but] these economic dimensions were often balanced against broader concerns with human rights and democracy.”^40^ However, the focus of the Conservative government (2006–2015) on the extractive sector generated much confusion regarding Canadian foreign aid and human rights policies in Latin America. Indeed, corporate interests took a predominant role “in shaping Canadian foreign policy in general and aid policy specifically.”^41^ Nevertheless, as Latin America was prioritized in Canadian foreign policy, LWBC’s capacity first grew exponentially during that period. Table 2

**Table 2.** Business and human rights issues during the Harper era (2006–2015).

Governing party	Context in Colombia	Canadian “security governance interventions”
Conservative government of Stephen Harper (2006–2015)	Canada-Colombia Free Trade Agreement signed on 21 November 2008 (comes into effect August 2011).	In 2007, the Harper government launches its “Strategy for the Americas” policy.
	Peace negotiations with FARC began in 2010 under President Juan Manuel Santos.	In 2009, the Government of Canada launches its first Corporate Social Responsibility (CSR) Strategy. In 2014, it announces an “enhanced” CSR Strategy.
		In 2010, support granted to LWBC in Colombia under START-GPSF.
		The Agreement Concerning Annual Reports on Human Rights and Free Trade Between Canada and the Republic of Colombia comes into effect on 15 August 2011.

In 2008, LWBC received a major programmatic funding from the Department of Foreign Affairs and International trade (DFAIT) for a new project on *Access to Justice for Indigenous Communities and Victims of Conflict*. While this project ostensibly reconciled human rights promotion and legal stability for business through the promotion of the rule of law, LWBC’s position on business and human rights issues in Colombia reflected the difficult conciliation between the two objectives. During this period, Canadian security governance interventions in Colombia were intrinsically linked with the negotiations of the Canada-Colombia Free Trade Agreement (CCOFTA).^42^ Appearing at the Standing Committee on International Trade in May 2008, LWBC’s director Pascal Paradis, emphasized that the NGO’s focus:

[…] is on the rule of law, justice and human rights, and it is our role to denounce human rights violations committed by a State. And if Canada embarks on free trade negotiations with that State, we believe it is our duty to urge caution.^43^

From 2010 to 2011, LWBC navigated the ambiguity within Canadian “security governance interventions” with some success. For example, they participated in an ONIC-organized Colombia-wide discussion on the right to prior consultation. Further, they monitored human rights violations when a Canadian mining company’s open-pit mining project in Marmato caused an increase in social conflict and displacements.^44^

Competing priorities, however, kept hindering Canada’s foreign policy, as illustrated by the elaboration of Annual Reports on Human Rights and Free Trade between Canada and the Republic of Colombia, an agreement in parallel with CCOFTA. These *ex-post* human rights impact assessments (HRIA) were criticized for their limited scope and methodology, which excluded the evaluation of the impacts of Canadian extractive investments on human rights.^45^ Although they first participated in these HRIAs, some non-governmental organizations (NGOs), such as Amnesty International, thus eventually decided to withdraw from the process “unless there [was] a substantial change in scope and methodology for the report.” They further stated that:

[…] the Government of Canada chooses to interpret its human rights reporting responsibilities in a limited and restrictive manner that ignores and overlooks pressing human rights concerns directly related to critical trade, investment and business policy and activities that are encouraged, promoted and furthered by the CCOFTA.^46^

In 2013, when CIDA and DFAIT were merged into one department, LWBC’s funding was reduced by 75 percent, which affected its ability to defend human rights.^47^

### Tiptoeing around the elephant in the room

The mandate of the Liberal government of Justin Trudeau in 2015 began with the intention of marking a clear difference from the Conservatives with regards to human rights in Latin America.^48^ This coincided with the conclusion of Colombia’s peace process and the implementation of the 2016 peace agreements. As illustrated in Table 3, LWBC’s projects over the last 5 years were supported by the Peace and Stabilization Operations Programs (PSOPs), Canada’s “principal platform for conflict prevention, stabilization and peacebuilding in fragile and conflict affected states.”^49^

**Table 3.** Transitional justice and Trudeau’s feminist agenda.

Governing party	Context in Colombia	Canadian “security governance interventions”
Liberal government of Justin Trudeau (2015–ongoing; data to 2021 only)	The Colombian government signs Peace Agreement with FARC in 2016.	Global Affairs Canada launches the Peace and Stabilization Operations Programs (PSOPs) in 2016, which replaces both START and GPSF.
	Election of President Iván Duque in 2018 and opposition to transitional justice institutions.	Canada announces additional support for peace implementation in Colombia in 2016.
	Attacks and killings of human rights defenders, social leaders, and former FARC-EP fighters after the Peace Agreement was signed.	Canada’s Feminist International Assistance Policy (FIAP) is introduced in 2017.
	Nationwide, anti-government protests begin on 28 April 2021; repression by security forces.	Canada launches Voices at Risk: Canada’s Guidelines on Supporting Human Rights Defenders in 2016.
		Canadian Ombudsperson for Responsible Enterprise (CORE) launches its human rights complaints process in 2021.

Due to the importance of Canada’s feminist foreign policy for the Trudeau government, LWBC’s new projects in Colombia were framed according to those goals and objectives. Since 2017, PSOPs have supported LWBC’s Transitional Justice for Women program (JUSTRAM) in Colombia, which aims to “actively foste[r] the participation of women and members of the Afro-Colombian and Indigenous communities in the national dialogue” and to promote “the emergence of a justice model that can inspire other States facing similar challenges.”^50^

The projects to enhance the rule of law have had a positive impact for human rights advocacy in Colombia; however, tension between the competing regional priorities of the Canadian government remains. While the Canadian embassy in Colombia “continued delivering upon its 2016 commitment of over $78 million in funding for peacebuilding efforts in Colombia,”^51^ none of the new projects involving LWBC directly address human rights violations linked to Canadian extractive companies, rather aligning with Canada’s commitment to corporate social responsibility (CSR).^52^

Organizations like LWBC creatively circumvent contradictions in Canada’s foreign policy objectives by using the apparent political neutrality of “the rule of law” to support important work. The requirements of transnational advocacy work, however, also produce “‘silences,’ exclusions, [and] paradoxical effects.”^53^ These “silences” are not trivial. As described in the next section, they may enable or facilitate attacks and stigmatization against human rights defenders accused of what Colombian army officials call “political warfare.”^54^

## “Libertad y Orden”: The politics of democracy and antipolitics in Colombia

Since 1834, Colombia’s motto has been “*Libertad y Orden,*” i.e*.*, “Liberty and Order.” Order and liberty, however, have often been at odds throughout its history.

This tension is reflected in two competing notions of the “rule of law.” For example, democratic and emancipatory regimes of reason in Colombia—whether liberally or radically inclined—associate the legitimacy of norms and laws with how they correspond to certain teleological aspirations. The rule of law, under this logic, is a means to an end. For example, the current Colombian Constitution enunciates what “the People of Colombia” consider to be the objectives of the Colombian Republic:

the unity of the Nation[,] the life of its members, peaceful coexistence, work, justice, equality, knowledge, freedom and peace[,] a legal, democratic and participatory framework that guarantees a just political, economic and social order, and [the promotion of] the integration of the Latin American community.^55^

The Constitution also ratifies—as does contemporary constitutionalism in general—“human dignity” and “human rights” as teleological aspirations.^56^ Under this logic, dissent and political conflict—as opposed to unanimity—are not only legitimate, but inherent to the expression of liberty.

Competing with this teleological logic is another regime of reason articulated around the imperative of “norm efficiency,” “stability,” or “order.” Under this logic, law is an end in itself: its legitimacy is a function of its ability to produce order, to rule, and to organize. This governmentality is, of course, reminiscent of Hobbesian philosophies; however, as Foucault’s genealogy of the notion of the “rule of law” highlights, the imperative of normative efficiency also underlies neoliberal condemnation of laws that interfere with a naturalized, self-enforcing “law” of the market.^57^ Under this form of governmentality, political dissent tends to be delegitimized as a factor of disorder or sub-optimal resource allocation. Consequently, conflict is reduced to its antagonistic dimension as inherently dangerous, rather than viewed through its agonistic, productive dimension.^58^ This opposing signification, which we call “antipolitical,” has considerable traction in a country ridden by poverty and by the longest armed conflict in the hemisphere.^59^

In the following sub-sections, we illustrate how these two governmentalities collide in two contemporary Colombian political sites: the intersection of counterinsurgency practices and transitional justice, and the instersection of territorial organization and natural resource management.

### Counterinsurgency practices, transitional justice, and the “rule of law”

“Hearts and Minds” counterinsurgency theories, originally developed by the French in Algeria, and updated by American theorists, have echoed throughout military doctrines adopted by Latin American armies from 1960 onward, notably in Colombia.^60^ These doctrines tend to adopt a singular understanding of the conflict, with a focus on the way that people think and feel. They also tend to view political dissent, albeit expressed through legal and democratic channels, as a challenge to the collective interest of the Nation, the integrity of which is embodied by the military.

These doctrines thus tend to substitute the classical (and legal) distinction between combatant and non-combatant, enemy and allied forces, with the criterion of ideological functionality: anyone who does not explicitly demonstrate full support for the counterinsurgency effort is to be considered functionally linked to the insurgency.^61^

The resulting rationality is antipolitical in the strictest sense: it pursues a cognitive and emotional control over society to prevent its members from organizing into a deliberative political community. In Colombia, for example, the construction of the internal enemy as a “terrorist[,] a moralizing process which imposes a norm on the whole of society,” is pervasive in official and paramilitary discourses.^62^ According to Colombian human rights defenders, the objective of this ideological construction is:

to infuse terror, punish and prevent political opposition or social claims, to deconstruct or break the communal bonds that serve as the basis for various forms of collective action [and] to exercise control over the population.^63^

While ostensibly pursuing the rule of law, Hearts and Minds doctrines necessarily uphold normative efficiency over teleological legitimacy. While General Ospina Valle describes the military’s aim as “democracy through security, but with a legitimate security concept limited by people’s rights,”^64^ he also affirms that *law—*and first and foremost *human rights law—*is “politicized” and “weaponized” to subvert the established order.^65^ For Brigadier General (ret’d) Fernando Puentes Torres, “political warfare”consists of attacking “the legitimacy of the State using the legitimate instruments of the State.”^66^ Indeed, according to the first French theoreticians of “political” or “irregular” warfare, if a country’s legal framework cannot be made explicitly counterinsurgent—by the traditional *coup d’état*, for example—then a degree of *illegality* in the fight against insurgency is necessary and inevitable.^67^ In Colombia, the “false positives scandal” provides a clear example of such malfeasance.^68^

The Colombian military intends to consolidate Hearts and Minds logics in the post-agreement era. Puentes Torres, who is also former executive director of military criminal justice, identifies “legal and judicial warfare” as “the most destructive” of “all the dimensions of political warfare” faced by the Colombian armed forces in the post-agreement era:^69^ “the worst of the war begins when […] those who defended their people and their homeland are tried without mercy.”^70^ Promoting this legal and judicial warfare are “illegal armed groups and related organizations (national or international NGOs as well as political parties linked to the extreme left) [who] rely on distorted, psychological and propagandistic actions.”^71^ These statements uphold the criteria of ideological functionality, blurring the distinction between an insurgent and someone whose actions might be useful to the insurgency while erasing any difference between legal and illegal military targets—a distinction, on the other hand, that is absolutely fundamental to democratic, constitutional, and teleological understanding of the rule of law. In Colombia, the antipolitical regime of reason clearly endangers human rights defenders, who have been victims of more than 450 killings since 2016, including ten assassinations of victims’ rights activists.^72^

It is at this point where coherence between human rights and legal certainty for investment reaches its limit. As previously mentioned, LWBC initially partnered with organizations directly involved in struggles concerning Indigenous people’s rights and extractivism, such as ONIC. One such organization, CAJAR, collaborated with other NGOs from the Working Group on Mining and Human Rights in Latin America to produce a report titled “The Impact of Canadian Mining in Latin America and Canada’s Responsibility,”^73^ which was submitted to the Inter-American Commission on Human Rights in 2013. The strictly legal activities of these organizations are vital to the full realization of constitutional and human rights. Nonetheless, the Colombian government has often stigmatized them as being functional to the guerillas, and as such, they have been victims of several attacks.^74^

This regime of reason also diverges from the transitional justice mechanisms put in place under the 2016 peace agreement. Indeed, opponents of the peace agreement perceive the Special Jurisdiction for Peace (JEP – *Jurisdicción Especial para la Paz*) as being too lenient on crimes committed by ex-FARC-EP fighters and too severe on military personnel accused of human rights violations. Former president Iván Duque’s (2018–2022) “emphasis on the rule of law has also gone hand in hand with denying the relevance and autonomy of the [JEP].”^75^ While Duque failed to undermine the JEP,^76^ the underlying antipolitical rationality remains a powerful driver of mobilization for certain elements of the state security apparatus and segments of Colombian society; the Colombian tradition of regionally based private armies and militarized economic elites has consequently been revived.^77^ In this context, Canada’s foreign policy cannot afford to be ambiguous about which rationality underlies the “rule of law” being put forward by different actors in post-agreement Colombia.

### Territorial organization, “legal stability,” and CSR

A parallel struggle is occurring at the intersection of resource extraction and territorial organization. At the heart of the struggle is the ongoing contradiction between the centralizing and technocratic tendencies of the Colombian elite and the constitutional principle of decentralized, “democratic, participatory [and] rational” territorial planning articulated around autonomous municipal governments.^78^ Between 1991 and the 2011 adoption of the first Land-Use Act (*Ley Orgánica de Ordenamiento Territorial, Ley 1454 de 2011*), the principle of democratic and decentralized land planning was left uncodified, which enabled a powerful central bureaucracy to impose its territorial planning on local administrations.^79^ Notwithstanding those impositions, social groups in various parts of Colombia have claimed this constitutional principle to empower popular democratic mobilization with the notable purpose of pre-empting or mitigating paramilitary control over local governments and territories.^80^ The struggle between these two logics—democratic and decentralized vs. technocratic and centralized—remains unresolved and regularly ends up in Colombia’s high courts.

Canada’s relationship with Colombia is inextricably linked to this struggle. Indeed, mineral extraction triggered a number of these court cases over the last 20 years,^81^ and Canada has supported Colombia in developing that sector for at least 25 years. According to James Rochlin, Canada had worked with the Colombian government “to create a mining program that was friendly to transnational investment”^82^ from 1997 until 2001. As a result, the 2001 Mining Code stipulates that “no regional, sectional or local authority may establish portions of their territories where mining activity would be temporarily or permanently prohibited.”^83^ The central government thus imposed the principle of “free mining”^84^ seeking to generate “legal stability” for mining title holders.

This extractivist rationality, which perceives the rule of law as securing normative stability for extractive investment, is clearly at odds with the realization of the constitutional principle of democratic and decentralized land planning.^85^ Indeed, the Mining Code has been successfully challenged in court for revoking municipal competence over territorial planning and for contradicting the decision-making rights of the local population with regard to land planning, notably through binding referenda on mining projects.^86^

These decisions, however, have not deterred the central government from upholding the principle of free mining.^87^ Echoing Canada’s position that human rights and a favourable investment environment in the extractive sector go hand in hand, the Colombian government has instead promoted corporation-community dialogue and corporate social responsibility agreements as a solution to conflicts between local communities and extractive operators. Colombia’s Business and Human Rights policy—“institutionalizing CSR under a rights-based approach”^88^—provides effective rhetorical support to Canada’s position by constructing a coherence between the management of “social and political risks” by private operators and the protection of human rights.^89^

Unfortunately, free mining is not so easily reconciled with human rights and the aspirations enshrined in Colombia’s constitution in practice. In fact, CSR displaces the overarching constitutional principle of decentralized and democratic land planning, and replaces it with negotiated forms of justice.^90^ In the process, local aspirations and grievances are reconstructed in a manner consistent with the extractive operator’s interests, which, in turn, are implicitly assumed to represent the interests of society as a whole.^91^ At its worst, the governmentality that emerges through this approach is one of a public-private Reason of State that is antagonistic to rights-based collective action. Under this governmentality, claims that are incompatible with the operators’ interests are stigmatized as contrary to the general interest of society, and complainants are often subjected to political violence.^92^

This dynamic is not exclusive to Colombia. In the Americas, extractive activities are often associated with unilateral affirmations of state sovereignty—over Indigenous title, for example. When challenged—whether through Indigenous claims to self-determination,^93^ alternative modes of territorial occupation, or development models incompatible with extractivism^94^—States tend to respond with charges of terrorism and other related accusations.^95^ Criminalization of dissent against the “commodities consensus”^96^ is thus a common issue related to extractive projects worldwide^97^ and especially in Latin America.^98^

CSR rationalities thus expand the counterinsurgent categories of the “internal enemy” that were outlined in the previous section. Indeed, territorial ordering structures an integral part of the post-agreement counterinsurgency doctrine championed by the Colombian military, which focuses on “civil society, territory and sovereignty.”^99^

The issue is particularly acute in Colombia. In fact, ongoing faith in Colombia’s peace process arguably hinges on successfully challenging the perpetuation of antipolitical counterinsurgency doctrines that provide “legal certainty” for Colombia’s land-owning economic elite and the extractive industry. The effects of these doctrines thus far are alarming. Since 2016, Colombians have witnessed the resurgence of “neo-paramilitary conservative forces that have taken over former FARC territories; a rise in killings of social leaders and ex-combatants […] and the often forceful pursuit of neoliberal reforms dispossessing marginalized populations of lands and livelihoods.”^100^ Clearly, an ambiguous stance in this context would only undermine Canada’s contribution to peace, human rights, and democracy in Colombia.

## Conclusion: Clarifying Canada’s position in Colombia

As the legacy of antipolitical counterinsurgency practices were internationally denounced and condemned during the April–March 2021 mobilizations in Colombia, there was something troubling about Canada’s official response to the crisis, in which police brutality and vandalism by protesters were put on an equal footing. In keeping with the two-faced nature of Canadian human rights policy in Latin America, the Canadian government positioned itself very carefully on both sides of the issue.

Less than 3 months later, on the 10th anniversary of the Canada-Colombia Free Trade Agreement on 14 July 2021, Canada’s minister of foreign affairs restated Canada’s commitment to “our ongoing partnerships in the areas of peace and security, human rights, democracy, trade and development.”^101^ As we have argued, the “weakness of the State” framework enables a convenient blurring of Canada’s contradicting interests in Colombia. Canada’s interventions to “strengthen the rule of law” have left the premise that there is a coherence between peace, human rights, and development unchallenged. However, failing to recognize the contradictions that may arise between these areas, depoliticizing the notion of “rule of law,” and minimizing crucial political tensions remains a hazardous strategy in post-agreement Colombia.

Despite considerable achievements made through strategic human rights litigation projects funded by the Canadian government in the last decade, including the invaluable work of LWBC, Canada’s security governance interventions in Colombia maintain blind spots on human rights issues related to transitional justice and extractivism.

Therefore, it seems important to pursue the analysis of the politicization and depoliticization of human rights issues through Canada’s interventions in post-conflict countries. How much more effective could these interventions be if the “weak State” framework was replaced with decisive support in favour of democratic conceptions of the rule of law and vigorous condemnation of anti-political rationalities.

## Notes

1. “Observations and recommendations: Working visit to Colombia,” Inter-American Commission on Human Rights, June 2021, https://www.oas.org/en/iachr/reports/pdfs/ObservacionesVisita_CIDH_Colombia_ENG.pdf (accessed 27 April 2022).

2. The Hon. Marc Garneau, “Canada concerned about persistent violence in Colombia,” Global Affairs Canada, 9 May 2021, https://www.canada.ca/en/global-affairs/news/2021/05/canada-concerned-about-persistent-violence-in-colombia.html (accessed 27 April 2022).

3. Christopher Campbell-Duruflé and Leila Celis, “With international pressure mounting on the Colombian government, Canada stands at a crossroads,” *The Conversation*, 14 July 2021, https://theconversation.com/with-international-pressure-mounting-on-the-colombian-government-canada-stands-at-a-crossroads-163042 (accessed 27 April 2022).

4. A decade ago, the Colombian State was described in the literature as “weak” as a result of corruption, war, and neoliberalism (Daron Acemoglu and James A. Robinson, *Why Nations Fail: The Origins of Power, Prosperity and Poverty* [New York: Crown Business, 2012], 70–123; the same concept has been employed with reference to the implementation of the 2016 peace agreement (Angelika Rettberg, “Peace-making amidst an unfinished social contract: The case of Colombia,” *Journal of Intervention and Statebuilding*, 14, no. 1 [2020]: 92).

5. David Carment and Yiagadeesen Samy, *Exiting the Fragility Trap: Rethinking our Approach to the World’s Most Fragile States* (Athens: Ohio University Press, 2019), 246.

6. Canada’s total official development assistance (ODA) to Colombia since 2011 is almost $497 million, which makes it Colombia’s fifth-largest donor after the United States, Germany, the European Union, and Norway. See “Annual Report Pursuant to the Agreement Concerning Annual Reports on Human Rights and Free Trade between Canada and the Republic of Colombia,” Embassy of Canada in Colombia, 2021, https://www.canadainternational.gc.ca/colombia-colombie/bilateral_relations_bilaterales/rep-hrft-co_2020-dple-rapp.aspx?lang=eng (accessed 27 April 2022).

7. “Annual Report,” Embassy of Canada in Colombia.

8. Our argument does not seek to disqualify civil society efforts articulated as revindications of the rule of law. Indeed, since the current Constitution was adopted in 1991, the language of human rights has been ever-present in the country’s political and legal arenas; marginalized groups have also won significant political battles partly by mobilizing the rhetoric of the rule of law, especially when pursuing cases in the Constitutional Court and the Inter-American Human Rights System (Centro Nacional de Memoria Histórica [CNMH], *El Derecho a La Justicia Como Garantía de No Repetición*, Bogotá, 2016); CNMH, Luz Marina Monzón, ed., *Balance de La Acción Del Estado Colombiano Frente a La Desaparición Forzada de Personas*, Tomo IV, 2014).

9. Jeffrey Monaghan, Security Aid: Canada and the Development Regime of Security (Toronto/Buffalo/London: University of Toronto Press, 2017), 4.

10. Ashraf Ghani and Clare Lockhart, Fixing Failed States: A Framework for Rebuilding a Fractured World (New York: Oxford University Press, 2009), 272.

11. For more nuanced critique of Canadian policy within the security-development nexus, see Stephen Baranyi, “Canada and the security-development nexus in Haiti: The ‘dark side’ or changing shades of gray,” Canadian Foreign Policy Journal 20, no. 2 (2014): 163–175.

12. Sonja Grimm, Nicolas Lemay-Hébert, and Olivier Nay, “‘Fragile States’: Introducing a political concept,” Third World Quarterly 35, no. 2 (2014): 202.

13. The 2020 FSI report states that “Colombia saw its worst year-on-year change in the history of the [FSI] in 2020, breaking a trend of almost uninterrupted improvement since the Index’s inauguration” (Charles Fiertz, “Colombia’s peace deal unravels,” Fragile States Index Annual Report, Washington: The Fund for Peace, 2020, 21, https://fragilestatesindex.org/wp-content/uploads/2020/05/fsi2020-report.pdf (accessed 27 April 2022).

14. Brad Amburn, “The Failed States Index 2005,” Foreign Policy, 22 October 2009, https://foreignpolicy.com/2009/10/22/the-failed-states-index-2005/ (accessed 27 April 2022).

15. Charles T. Call, “The fallacy of the ‘failed state,’” Third World Quarterly 29, no. 8 (2008): 1491–1507.

16. Jeremy Farrall and Hilary Charlesworth, eds., Strengthening the Rule of Law through the UN Security Council (London: Routledge, 2016), 328.

17. Michel Foucault, Naissance de la biopolitique: Cours au Collège de France (1978–1979) (Paris: Gallimard/Seuil, 2004), 79.

18. Ibid., 335; see also Michel Foucault, Le gouvernement de soi et des autres: Cours au Collège de France 1982–1983 (Paris: Gallimard/Seuil: 2008), 6.

19. Susan Strange, States and Markets (London: Bloomsbury Academic, 1998), 304.

20. David Chandler, “New narratives of international security governance: The shift from global interventionism to global self-policing,” Global Crime 17, no. 3–4 (2016): 264.

21. David Chandler, “The security–development nexus and the rise of ‘anti-foreign policy,’” Journal of International Relations and Development 10, no. 4 (2007): 379.

22. Monaghan, Security Aid, 31.

23. Jacqueline Best analyzes the role of ambiguity as an *object* of global governance, a *strategic asset* in governance, and a *limit* to governance: Jacqueline Best, “Ambiguity, uncertainty and risk: Rethinking indeterminacy,” International Political Sociology, 2 no. 4 (2008), 355–374. See also Charles Saliba-Couture, Les liens entre sécurité et développement: De l’évidence à l’ambiguïté (Paris: L’Harmattan, 2012), 242.

24. Stephen Brown, “A Samaritan state?, Canadian foreign aid, and the challenges of policy coherence for development,” in Greg Donaghy and David Webster, eds., A Samaritan State Revisited: Historical Perspectives on Canadian Foreign Aid (Calgary: University of Calgary Press, 2019), 311–329.

25. For example, the controversy between international realists and humane internationalists; see David R. Black, “Humane internationalism and the malaise of Canadian aid policy,” in Stephen Brown, Molly den Heyer, and David R. Black, eds., Rethinking Canadian Aid, 2nd ed. (Ottawa: University of Ottawa Press, 2016), 17–33.

26. Ibid., 18–23.

27. Laura Macdonald, “Canadian development assistance to Latin America,” in Greg Donaghy and David Webster, eds., A Samaritan State Revisited: Historical Perspectives on Canadian Foreign Aid (Calgary: University of Calgary Press, 2019), 274.

28. “Canada-Colombia relations,” Global Affairs Canada, 26 November 2021, https://www.international.gc.ca/country-pays/colombia-colombie/relations.aspx?lang=eng (accessed 27 April 2022). See also “Rule of Law,” Government of Canada, 6 May 2017, https://www.international.gc.ca/world-monde/issues_development-enjeux_developpement/human_rights-droits_homme/rule_law-primaute_droit.aspx?lang=eng (accessed 27 April 2022).

29. See Marc-André Anzueto, “De la responsabilité sociale des entreprises à la conviction responsable: les politiques canadiennes de droits humains en Colombie et au Guatemala (2015–2019),” Études canadiennes 89 (2020): 205–231.

30. Marc-André Anzueto, “In the shadow of Canadian imperialism? Strategic human rights litigation in Guatemala (2009–2019),” The International Journal of Human Rights 25, no. 10 (2021): 1670–1671.

31. Pablo Heidrich, “Determinants, boundaries, and patterns of Canadian mining investments in Latin America (1995–2015),” Latin American Policy 7, no. 2 (2016): 204.

32. Canadian mining assets: Information bulletin,” Natural Resources Canada, 21 February 2022, https://www.nrcan.gc.ca/maps-tools-and-publications/publications/minerals-mining-publications/canadian-mining-assets/19323#F2 (accessed 27 April 2022); “Canadian mining assets (CMA) by country and region, 2019 and 2020,” Natural Resources Canada, 21 February 2022, https://www.nrcan.gc.ca/maps-tools-and-publications/publications/minerals-mining-publications/canadian-mining-assets/canadian-mining-assets-cma-country-and-region-2018-and-2019/15406 (accessed 27 April 2022).

33. Shin Imai, Leah Gardner, and Sarah Weinberg, “The ‘Canada brand’: Violence and Canadian mining companies in Latin America,” Osgoode Legal Studies Research *Paper no. 17* (2017): 12, 83–87.

34. Stephen Brown, “Foreign aid, the mining sector and democratic ownership: The case of Canadian assistance to Peru,” Development Policy Review 38, no. 51 (2020): 15.

35. According to its 2020–2021 annual report, LWBC had seven offices worldwide, employing 109 people with ongoing projects in Colombia, Haiti, Honduras, El Salvador, Mali, and Guatemala. The new strategic plan for 2021–2024 plans “to consolidate LWBC’s position as an indispensable actor in legal empowerment, in Canada and around the world.” See “Annual Report 2020–2021: Taking pride in commitment,” Lawyers Without Borders Canada, 2021, https://www.asfcanada.ca/rapport2020-2021/en/ (accessed 27 April 2022).

36. Anzueto, “De la responsabilité sociale des entreprises,” 222.

37. Activities Report: From the 1st of January 2008 to the 30th of June 2009,” Lawyers Without Borders, Québec, 4 Septmeber 2009, https://www.asfcanada.ca/uploads/publications/uploaded_rapport-d-activites-asfc-2008-09-pdf-3.pdf (accessed 27 April 2022).

38. Canada, Parliament, House of Commons, Comité permanent du commerce international, *Témoignage d’Avocats sans frontières Canada*, 2nd sess. 39th Parliament, Meeting of 26 May 2008, https://www.asfcanada.ca/uploads/publications/uploaded_presentation-d-asf-au-cpci-26-mai-2008-pdf-14.pdf (accessed 27 April 2022).

39. “Evaluation of the Stabilization and Reconstruction Task Force (START) and Global Peace and Security Fund (GPSF),” Global Affairs Canada, Ottawa, 2016, https://www.international.gc.ca/gac-amc/publications/evaluation/2018/start-gpsf.aspx?lang=eng (accessed 27 April 2022).

40. Macdonald, “Canadian development assistance to Latin America,” 285.

41. Ibid., 285.

42. Canada’s free trade negotiations with Colombia date back to 2002 when Canada first held exploratory discussions on a possible free trade agreement with the members of the Andean Community (Peru, Colombia, Ecuador, and Bolivia). See “Human rights, the environment and free trade with Colombia: Report of the Standing Committee on International Trade,” House of Commons Canada, June 2008, https://www.ourcommons.ca/Content/Committee/392/CIIT/Reports/RP3580301/ciitrp05/ciitrp05-e.pdf (accessed 27 April 2022).

43. Comité permanent du commerce international, *Témoignage d’Avocats sans frontières Canada*.

44. See “Rapport d’activités 2010–2011, Pour que justice soit faite,” Lawyers Without Borders Canada, 2011 https://www.asfcanada.ca/uploads/publications/uploaded_asf-rapport-annuel-2010-2011-web-pdf-18.pdf (accessed 27 April 2022).

45. See Etienne Roy Grégoire, “Les rapports annuels sur les impacts de l’ALE Canada-Colombie sur les droits humains au regard des enjeux entourant l’investissement extractif en Colombie: limites méthodologiques, crédibilité et pertinence,” Annuaire Canadien des Droits Humains, no. 1 (2017): 19–28.

46. Alex Neve and Geneviève Paul, “Letter to Global Affairs Canada on the Annual Report on Human Rights and Free Trade Between Canada and the Republic of Colombia,” Amnesty International, 20 March 2018, https://www.amnesty.ca/sites/amnesty/files/LetterToGAC_CCOFTAhumanrightsReport2018-fromAmnestyInternational%281%29.pdf (accessed 27 April 2022).

47. Anzueto, “In the shadow of Canadian imperialism,” 1670.

48. Anzueto, “De la responsabilité sociale des entreprises,” 218–219.

49. “Peace and Stabilization Operations Program,” Global Affairs Canada, 2022, https://www.international.gc.ca/world-monde/issues_development-enjeux_developpement/response_conflict-reponse_conflits/psop.aspx?lang=eng (accessed 27 April 2022).

50. “Colombia Projects: Transitional Justice for Women (JUSTRAM),” Lawyers Without Borders Canada, 2021, https://www.asfcanada.ca/en/projet/projets-en-cours/cooperation-internationale-droits-des-femmes-colombie-asfcanada/ (accessed 27 April 2022).

51. “Annual Report,” Embassy of Canada in Colombia.

52. Anzueto, “De la responsabilité sociale des entreprises,” 220.

53. Kathryn Sikkink, “Transnational advocacy networks and the social construction of legal rules,” in Yves Dezalay and Bryan G. Garth, eds., Global Prescriptions: The Production, Exportation, and Importation of a New Legal Orthodoxy (Michigan: University of Michigan Press, 2002), 56.

54. See Brigadier General (ret’d) Fernando Puentes Torres, “The continuous judicial and the judiciary wars in Colombia: Critical dimensions of insurgent political warfare,” Journal of Military and Strategic Studies, 17, no. 4 (2017): 247–253.

55. Constitución Política de Colombia, Preámbulo.

56. Constitución Política de Colombia, title II. chap. VI. art. 93.

57. Foucault, Naissance de la biopolitique, 368.

58. Chantal Mouffe, Agonistics: Thinking The World Politically (London & New York: Verso, 2013), 224.

59. Pablo Emilio Angarita Cañas et al., *La construcción del enemigo en el conflicto armado colombiano 1998–2010* (Medellín: Sílaba Editores, Instituto de Estudios Region-ales, Universidad de Antioquia, 2015), 316.

60. Philippe Dufort, “A typology of military factions in the Colombian officer corps: Origins and evolution of Colombian counter-insurgency,” Canadian Journal of Latin American and Caribbean Studies 42, no. 3 (2017): 327–341.

61. Marc Drouin, “‘Permitir que solamente la buena planta crezca’: La Guerra contrasubversiva guatemalteca y sus raíces francesas,” in Roberto García Ferreira and Arturo Taracena, eds., *La Guerra Fría y el Anticomunismo en Centroamérica* (Ciudad Guatemala: FLACSO Guatemala, 2017), 196–198; Alexander Grenoble and William Rose, “David Galula’s counterinsurgency: Occam’s Razor and Colombia,” Civil Wars 13, no. 3 (2011): 284–286.

62. Angarita Cañas et al., La construcción del enemigo, 83, 204.

63. Equipo interdisciplinario ad hoc sobre impunidad y memoria, eds., *Memoria de la impunidad en Antioquia: Lo que la justicia no quisó ver frente al paramilitarismo* (Medellín: Instituto Popular de Capacitación and Corporación Jurídica Libertad, 2010), 10.

64. General (ret’d) Carlos Ospina Ovalle, “Legitimacy as the center of gravity in hybrid warfare: Notes from the Colombian battlefield,” Journal of Military and Strategic Studies 17, no. 4 (2017): 258.

65. Ibid., 258.

66. Puentes Torres, “The continuous judicial and the judiciary wars in Colombia,” 247.

67. Drouin, “‘Permitir que solamente la buena planta crezca,’” 204.

68. In Colombia, the term “false positives” refers to “the systematic killing of socio-economically disadvantaged civilians by the military forces of Colombia and associated groups, particularly between 2002 and 2008, presented as guerrilla fighters that have been lawfully killed in combat” (Eleanor Gordon et al. “Power, poverty and peacebuilding: The violence that sustains inequalities and undermines peace in Colombia,” Conflict, Security & Development 20, no. 6 [2020]: 698). More recently, the Colombian government partially acknowledged its responsibility in a case of “false positives” before the Inter-American Court of Human Rights (see, for example, Villamizar Durán et al. *v.* Colombia. 2018-C364 IACt H.R., 20 November 2018).

69. Puentes Torres, “The continuous judicial and the judiciary wars in Colombia,” 249.

70. Ibid., 253.

71. Ibid., 248–249.

72. José Miguel Vivanco, “Amicus brief on killings of human rights defenders in Colombia,” Human Rights Watch, 20 April 2021, https://www.hrw.org/news/2021/04/20/amicus-brief-killings-human-rights-defenders-colombia (accessed 27 April 2022).

73. “The impact of Canadian mining in Latin America and Canada’s responsibility: Executive summary of the report submitted to the Inter-American Commission on Human Rights,” Due Process of Law Foundation (DPLF), 2014, 40, http://www.dplf.org/sites/default/files/report_canadian_mining_executive_summary.pdf (accessed 27 April 2022).

74. In 2020, prior to the 2021 strike, mass protests and disproportionate use of force by police officers in Colombia, twenty national and regional Colombian organizations, including CAJAR and ONIC, presented a document coordinated by the Comisión Colombiana de Juristas (2020) entitled *El Riesgo de Defender y Liderar* [*The Risk of Defending and Leading*], which contributed to the denunciation and visibility of common patterns and differential effects of rights violations against human rights defenders in Colombia. See “El riesgo de defender y liderar: Pautas comunes y afectaciones diferenciales en las violaciones de los derechos humanos de las personas defensoras en Colombia, Comisión Colombiana de Juristas,” 2020, https://www.coljuristas.org/documentos/libros_e_informes/InformeElriesgodedefenderyliderarDEF.pdf (accessed 27 April 2022).

75. Giulia Piccolino and Krisna Ruette-Orihuela, “The turn from peacebuilding to stabilisation: Colombia after the 2018 presidential election,” Third World Quarterly 42, no. 10 (2021): 2401.

76. Adrián Raúl Restrepo Parra, “El gobierno desestabilizador del Acuerdo,” *Revista Debates* 81, Especial-JEP (2019): 26–33.

77. Dufort, “A typology of military factions,” 342.

78. Colombian Constitutional Court, “Sentencia de Constitucionalidad no. 006-02 de Corte Constitucional,” 23 January 2002, https://www.corteconstitucional.gov.co/relatoria/2002/C-006-02.htm (accessed 25 August 2022).

79. Gustavo Zafra Roldán, “Antioquia: Precursora de la autonomía territorial,” in Hernando Yepes Arcila and Vanessa Suelt Cock, eds., *La Constitución, 20 años después. Visiones desde la teoría y la práctica constitucional* (Bogotá: Grupo Editorial Ibañez, Pontifica Universidad Javeriana, Centro de Estudios Constitucionales, 2012), 453.

80. Sandra Mildred Hincapié Jiménez, Democracia y poder constituyente: movimiento constituyente de Antioquia (Medellín: Instituto Popular de Capacitación [IPC], 2008).

81. See, for example, the following decisions in Colombia’s Constitutional Court: *Sentencia C-366 de 2011*, 11 May 2011; *Sentencia T-204 de 2014*, 27 January 2014; *Sentencia C-273 de 2016*, 25 May 2016; *Sentencia T-445 de 2016*, 19 August 2016; *Sentencia SU-095 de 2018*, 11 October 2018.

82. James Rochlin, “Informal gold miners, security and development in Colombia: Charting the way forward,” The Extractive Industries and Society 5, no. 3 (2018): 333.

83. Por la cual se expide el Código de Minas y se dictan otras disposiciones, Ley 685 de 2001, el Congreso de Colombia, s. 37 (2001).

84. Myriam Laforce, Véronique Lebuis, and Ugo Lapointe, “Régulation du secteur minier au Québec et au Canada. Une redéfinition des rapports asymétriques est-elle possible?” in Myriam Laforce, Bonnie Campbell, and Bruno Sarrasin, eds., Pouvoir et régulation dans le secteur minier. Leçons à partir de l’expérience canadienne (Laval: Presses de l’Université du Québec, 2012), 9.

85. Etienne Roy Grégoire, *Écosystèmes normatifs miniers et communautés politiques en Colombie transitionelle*, doctoral thesis, Université d’Ottawa, 2019.

86. See Osorio Jaramillo y otros v. *Colombia*, Sentencia C-273 de 2016, Corte Constitucional (2016), and *Flores Arcila v. el Tribunal Administrativo del Quindío*, Sentencia T-445 de 2016, Corte Constitucional (2016), 164.

87. See Kristina Dietz, “Direct democracy in mining conflicts in Latin America: Mobilising against the La Colosa project in Colombia,” Canadian Journal of Development Studies / Revue canadienne d’études du développement 40, no. 2, (2017): 145–162.

88. “Plan Nacional de Acción de Empresas y Derechos Humanos 2020–2022 ‘Juntos Lo Hacemos Posible: Resiliencia y Solidaridad,’” Consejería Presidencial para los derechos humanos y asuntos internacionales (CPDDHAI), 2020, http://www.derechoshumanos.gov.co/Prensa/2020/Documents/Plan-Nacional-de-Accion-de-Empresa-y-Derechos-Humanos.pdf (accessed 27 April 2022).

89. See James Rochlin, “A golden opportunity lost: Canada’s human rights impact assessment and the free trade agreement with Colombia,” The International Journal of Human Rights 18, no. 4–5 (2014): 545–566. See also Rochlin, “Informal goldminers,” 330–339.

90. David Szablowski, “Operationalizing free, prior, and informed consent in the extractive industry sector? Examining the challenges of a negotiated model of justice,” Canadian Journal of Development Studies/Revue canadienne d’études du développement 30, no. 1 (2010): 111–130. In this regard, Colombia is a good illustration of a global shift toward governance prescriptions that favour reflexive and self-enforcing norms, like CSR, in response to a perceived inefficiency of law-like norms to tackle business and human rights issues. See Thomas Berns, “L’efficacité comme norme,” *Dissensus 4* (2011); also Penelope Simons and Audrey Macklin, *The Governance Gap: Extractive Industries, Human Rights, and the Home State Advantage*, (London: Taylor & Francis Group, 2014): 12–16.

91. Thomas Berns, “Si les entreprises ont une âme,” in Thomas Berns, Pierre-François Docquir, Benoît Frydmùan, Ludovic Hennebel, and Gregory Lewkowicz, eds., *Responsabilités des entreprises et corégulation* (Brussels: Bruylant 2001), 51–78. President Juan Manuel Santos justified the Peace Process with the FARC-EP by equating “peace” with the continuity of a neoliberal economic model (Philippe Le Billon, María Cecilia Roa-García, and Angelica Rocío López-Granada, “Territorial peace and gold mining in Colombia: Local peacebuilding, bottom-up development and the defence of territories,” *Conflic*t, Security & Development 20, no. 3 (2020); John-Andrew McNeish, “Extracting justice? Colombia’s commitment to mining and energy as a foundation for peace,” The International Journal of Human Rights 21, no. 4 [2017]: 500–516) and specifically argued that “mining [was] the engine that would pull the Peace train.” See “Prosperidad Para Todos: Más Em-pleo, Menos Pobreza y Más Seguridad – Bases Del Plan Nacional de Desarrollo 2011–2014,” República de Colombia, Departamento Nacional de Planeación, (2011), https://colaboracion.dnp.gov.co/CDT/PND/BasesPND2010-2014Versi%C3%B3n514-04-2011completo.pdf (accessed 27 April 2022). Despite divergences between President Duque and former President Juan Manuel Santos’ approaches towards Colombia’s peace-building process, the Colombian government still embraces extractivism.

92. See Etienne Roy Grégoire and Luz Marina Monzón, “Institutionalising CSR in Colombia’s extractive sector: Disciplining society, destabilising enforcement?” Canadian Journal of Development Studies / Revue canadienne d’études du développement 38, no. 2 (2017): 253–271.

93. Inter-American Commission on Human Rights, Pueblos indígenas, comunidades afrodescendientes y recursos naturales: Protección de derechos humanos en el contexto de actividades de extracción, explotación y Desarrollo (Washington, DC: CIDH, 2016), 183.

94. See Arnim Scheidel, Daniela Del Bene, Juan Liu, Grettel Navas, Sara Mingorría, Federico Demaria, Sofía Avila, Brototi Roya, Irmak Ertörac, Leah Temper, and Joan Martínez-Aliera, “Environmental conflicts and defenders: A global overview,” Global Environmental Change 63 (July 2020): 1–12.

95. “In the national interest? Criminalization of environmental and human rights defenders in the Americas,” MiningWatch Canada and the International Civil Liberties Monitoring Group (ICLMG), Ottawa, 2015, https://iclmg.ca/wp-content/uploads/2015/09/IntheNationalInterest_FullReport.pdf (accessed 27 April 2022).

96. See Maristella Svampa, “Commodities consensus: Neoextractivism and enclosure of the commons in Latin America,” South Atlantic Quarterly 114, no. 1 (2015): 65–82.

97. See Scheidel et al., “Environmental conflicts and defenders,” 1–12.

98. Inter-American Commission on Human Rights, Criminalización de la labor de las defensoras y los defensores de derechos humanos (Washington, DC: CIDH, 2015), 164.

99. Ospina Ovalle, “Legitimacy as the center of gravity,” 256.

100. Philippe Le Billon, María Cecilia Roa-García, and Angelica Rocío López-Granada, “Territorial peace and gold mining in Colombia: Local peacebuilding, bottom-up development and the defence of territories,” Conflict, Security & Development 20, no. 3 (2020): 304.

101. “Minister Garneau speaks with the Vice President and Foreign Affairs Minister of Colombia,” Global Affairs Canada, 14 July 2021, https://www.canada.ca/en/global-affairs/news/2021/07/minister-garneau-speaks-with-the-vice-president-and-foreign-affairs-minister-of-colombia.html (accessed 27 April 2022).

